# A rare cause of acute upper airway obstruction: a case report

**DOI:** 10.1186/s12245-025-00908-1

**Published:** 2025-07-31

**Authors:** Jane Harding, Csaba Dioszeghy

**Affiliations:** Surrey and Sussex Healthcare NHS Trust, East Surrey Hospital, Redhill, Surrey UK

**Keywords:** Acute airway obstruction, Achalasia, Oesophageal dilation, Case report

## Abstract

**Background:**

Sudden collapse and fluctuating consciousness in elderly patients has a broad differential diagnosis, yet timely diagnosis can be critical for appropriate management. This case report underscores the importance of considering uncommon etiologies in patients presenting with such nonspecific symptoms.

**Case presentation:**

An 82-year-old female presented to the Emergency Department following a collapse at home and reduced conscious level associated with episodes of respiratory arrest. Computed Tomography (CT) pulmonary angiography revealed a significantly dilated oesophagus with tracheal compression. Following successful intubation and identification of the dilated oesophagus with tracheal compression, the patient’s condition stabilized and she was later successfully extubated and discharged from hospital.

**Conclusions:**

This report adds to the literature on uncommon causes of acute respiratory distress and emphasises the importance of maintaining a broad differential diagnosis when evaluating elderly patients presenting with sudden collapse and respiratory symptoms.

## Background

Sudden collapse and fluctuating consciousness in elderly patients are symptoms that can arise from a myriad of causes, ranging from cardiovascular events, such as strokes and heart attacks, to metabolic imbalances and neurological conditions. The differential diagnosis for these symptoms is broad, making timely and accurate diagnosis challenging yet critical for effective management. This case report underscores the importance of considering uncommon aetiologies in patients presenting with such nonspecific symptoms.

This case report presents an 84-year-old woman with no significant past medical history who presented with sudden onset of symptoms, including collapse and fluctuating conscious level, which initially led to a suspicion of stroke, highlighting the importance of thorough evaluation and differential diagnosis in such cases.

## Case presentation

An 82-year-old female patient, who was previously healthy, independent, and living with her husband, was presented to the Emergency Department (ED) by ambulance after a sudden collapse at home. On arrival of the paramedics, the patient was witnessed to have a fluctuating conscious level, with periods of unconsciousness accompanied by episodes of respiratory arrest and periods of sudden return of consciousness associated with severe agitation. The paramedics alerted the ED of a suspected stroke.

Upon arrival, the patient was responsive but extremely agitated and showed signs of respiratory distress. Her oxygen saturation was 88% on 15 L of supplemental oxygen via a face mask. Her respiratory rate was 30/min, heart rate was 116/min, non-invasive blood pressure was 176/90 mmHg, and Glasgow Coma Scale (GCS) score was 12/15 (E4 V2 M6). A quick physical examination revealed central cyanosis and diaphoretic skin, but no audible stridor or wheezing. On auscultation, she had bilateral equal chest expansion with minimally wheezy and reduced breath sounds bilaterally. The patient was agitated and unable or unwilling to speak but moved all limbs symmetrically without any obvious signs of lateralisation.

Her bedside arterial blood gas (ABG) results showed a respiratory acidosis (pH: 7.07, pCO2:11.8 kPa, PO2:11.1 kPa on 85% FiO2, BE: −2, lactate 2.5 mmol/L, and blood glucose 18.4 mmol/L). Point-of-Care Ultrasound (POCUS) showed no signs of pneumothorax or B lines, with good left ventricular function and no dilated right ventricle.

The initial presentation with the paramedics suggested a possible stroke, but clinical examination and diagnostic tests on arrival at the Emergency Department indicated an acute respiratory issue. The ABG results indicated severe respiratory acidosis. Bedside ultrasound ruled out pneumothorax, atelectasis, acute left ventricular failure, and massive pulmonary embolism (PE); however, smaller PE was still possible. Although an acute asthma attack without a previous history was rather unlikely, we had very limited data about past medical history at this time; therefore, the initial differential diagnosis included severe acute asthma with silent chest based on physical examination findings.

Initial interventions included oxygen therapy and attempts to treat a potential severe acute asthma attack with nebulised beta-mimetic and adrenaline. As the bedside echocardiography ruled out acute right ventricular failure as a sign of massive PE, thrombolysis was not indicated. When the initial treatments proved ineffective, upper airway obstruction due to foreign body inhalation was considered, and rapid sequence intubation (RSI) was performed. Intubation was successful, with the endotracheal tube passing into the trachea without difficulty, resulting in immediate improvement in oxygenation and bilateral breath sounds. During laryngoscopy, intubation and the positive pressure ventilation there was no sign of any foreign body in the upper or lower airways. Capnography confirmed the tube position and revealed a normal curve without signs of increased airway resistance. The ventilation pressures (Peak Inspiratory and Inspiratory Plateau Pressures) were normal, indicating normal lung compliance. Repeated arterial blood gas analysis showed normal oxygenation and ventilation parameters.

**Fig. 1:**
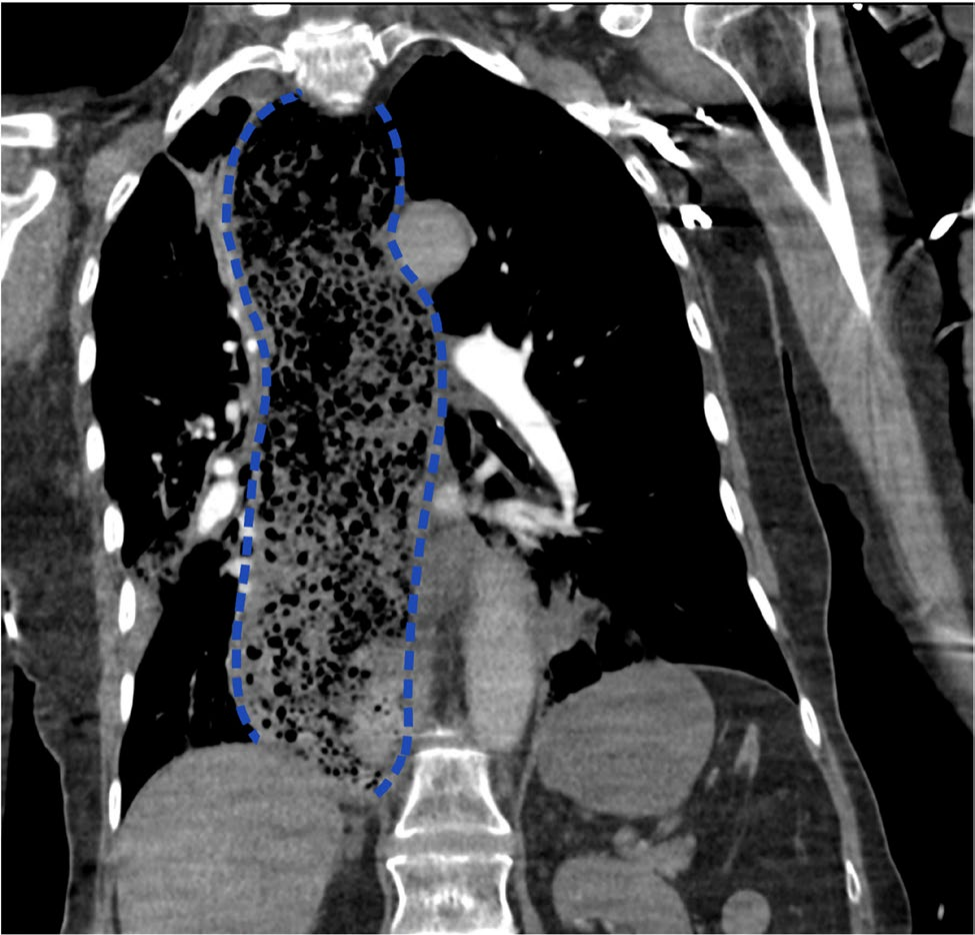
Coronal view of CT pulmonary angiogram taken on admission showing a grossly dilated oesophagus with extensive food residue, highlighted in blue

**Fig. 2:**
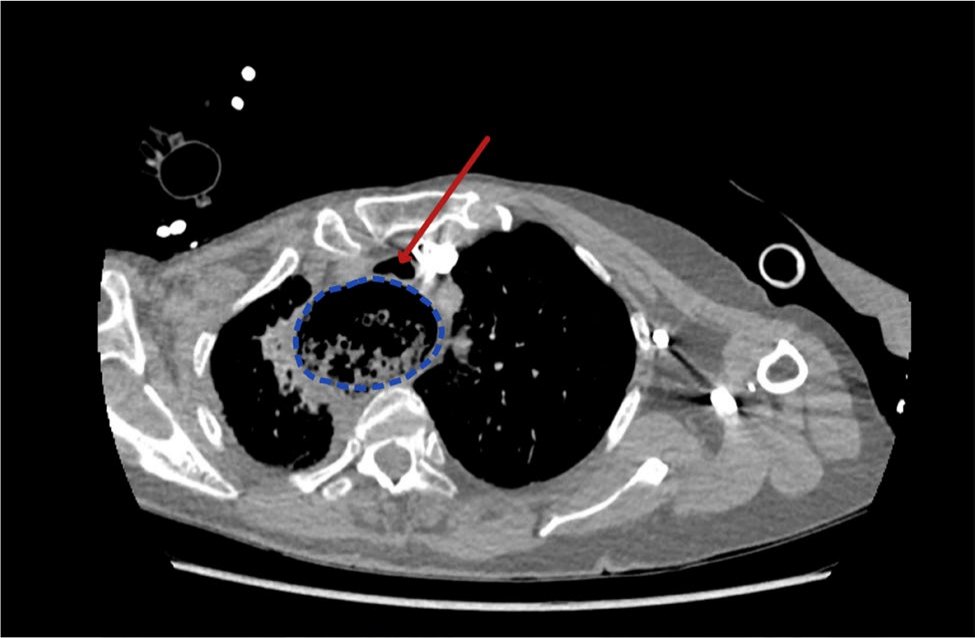
Axial view of CT pulmonary angiogram taken on admission showing a grossly dilated oesophagus (circled in blue) with compression of the trachea (highlighted by red arrow)

After RSI, CT pulmonary angiography was performed to rule out non-massive PE, which revealed a significantly dilated oesophagus with tracheal compression, filled with food residue, without an abrupt transition point, as seen in Figs. 1 and 2.

Following successful intubation and identification of the dilated oesophagus with tracheal compression, the patient’s condition stabilised. After nasogastric tube insertion, the patient was transferred to the Critical Care Department, where a subsequent oesophago-gastro-duodenoscopy (OGD) confirmed the diagnosis. She was successfully extubated 8 days later and discharged from the hospital after 20 days with appropriate follow-up.

## Discussion

Achalasia is a rare oesophageal motility disorder characterised by impaired relaxation of the lower oesophageal sphincter and loss of peristalsis in the oesophageal body [1]. The aetiology of achalasia is not fully understood, but it is believed to result from the degeneration of inhibitory neurons in the myenteric plexus of the oesophagus [1, 2].

The prevalence of achalasia is estimated to be approximately 10 per 100,000 individuals, with an estimated annual incidence of 1 per 100,000 people [3].

In severe cases, progressive dilation of the oesophagus can lead to complications such as tracheal compression. Although rare, this complication can cause significant respiratory distress and, in extreme cases, can be fatal. The incidence of tracheal compression due to achalasia is not well documented, but it is considered an uncommon manifestation of advanced disease, typically occurring in long-standing, untreated cases or in patients with a mega-oesophagus [4–7]. To date, case reports have predominantly described elderly female patients presenting with acute symptoms following a meal [7]. A previous medical history of achalasia, clinical signs of stridor, and choking episodes during a meal often direct clinicians to airway obstruction. However, in the rare presentation without obvious stridor, the initial differential diagnosis often includes acute asthma due to the silent chest presentation [8]. Radiology is the most common diagnostic modality with subsequent endoscopic confirmation [9].

In our case, past medical history or more details related to the circumstances of the collapse was lacking, and the only information received from the paramedics was that the collapse occurred some short time after a large meal with the family. No further information was available at the time and the patient was not able to communicate. The following day we discovered from speaking to her next-of-kin that the patient had an approximately 2-month history of feeling like food was being stuck after eating, although this information was not initially presented to our team.

Immediate treatment aims to secure the airway. Oesophageal decompression via a nasogastric tube is often possible, but endotracheal intubation may be necessary in rare cases [10].

## Conclusion

This case report presents an unusual cause of acute respiratory distress in an elderly patient, where a dilated oesophagus led to tracheal compression. This case emphasises the importance of maintaining a broad differential diagnosis, even when the initial presentation suggests more common conditions. Prompt recognition and appropriate management, including airway securing, were crucial for stabilising the patient’s condition. This report adds to the literature on rare causes of acute respiratory distress and highlights the need for a thorough evaluation of elderly patients presenting with sudden collapse and respiratory symptoms.

## Data Availability

No datasets were generated or analysed during the current study.

## Abbreviations

ABG: Arterial blood gas
CT: Computed tomography
GCS: Glasgow coma scale
OGD: Oesophago–gastro–duodenoscopy
PE: Pulmonary embolism
POCUS: Point–of–care ultrasound
RSI: Rapid sequence induction
